# Unveiling Sociocultural Barriers to Breast Cancer Awareness Among the South Asian Population: Case Study of Bangladesh and West Bengal, India

**DOI:** 10.2196/53969

**Published:** 2025-01-10

**Authors:** Fahmida Hamid, Tania Roy

**Affiliations:** 1 New College of Florida Sarasota, FL United States

**Keywords:** Bangladesh, West Bengal, India, Asia, breast cancer, awareness, early detection, screening, sociocultural barriers, health knowledge, cultural, stigma, social media, socioeconomic

## Abstract

**Background:**

Bangladesh and West Bengal, India, are 2 densely populated South Asian neighboring regions with many socioeconomic and cultural similarities. In dealing with breast cancer (BC)–related issues, statistics show that people from these regions are having similar problems and fates. According to the Global Cancer Statistics 2020 and 2012 reports, for BC (particularly female BC), the age-standardized incidence rate is approximately 22 to 25 per 100,000 people, and the age-standardized mortality rate is approximately 11 to 13 per 100,000 for these areas. In Bangladesh, approximately 90% of patients are at stages III or IV, compared with 60% in India. For the broader South Asian population, this figure is 16%, while it is 11% in the United States and the United Kingdom. These statistics highlight the need for an urgent investigation into the reasons behind these regions’ late diagnoses and treatment.

**Objective:**

Early detection is essential for managing BC and reducing its impact on individuals. However, raising awareness in diverse societies is challenging due to differing cultural norms and socioeconomic conditions. We aimed to interview residents to identify barriers to BC awareness in specific regions.

**Methods:**

We conducted semistructured interviews with 17 participants from West Bengal and Bangladesh through Zoom (Zoom Video Communications). These were later transcribed and translated into English for qualitative data analysis. All our participants were older than 18 years, primarily identified as female, and most were married.

**Results:**

We have identified 20 significant barriers to effective BC care across 5 levels—individual, family, local society, health care system, and country or region. Key obstacles include neglect of early symptoms, reluctance to communicate, societal stigma, financial fears, uncertainty about treatment costs, inadequate mental health support, and lack of comprehensive health insurance. To address these issues, we recommend context-specific solutions such as integrating BC education into middle and high-school curricula, providing updates through media channels like talk shows and podcasts, promoting family health budgeting, enhancing communication at cultural events and religious gatherings, offering installment payment plans from health care providers, encouraging regular self-examination, and organizing statewide awareness campaigns. In addition, social media can be a powerful tool for raising mass awareness while respecting cultural and socioeconomic norms.

**Conclusions:**

Fighting BC or any fatal disease is challenging and requires support from various dimensions. However, studies show that raising mass awareness is crucial for the early detection of BC. By adopting a sensitive and well-informed approach, we aim to improve the early detection of BC and help reduce its impact on South Asian communities.

## Introduction

According to the GLOBOCAN (Global Cancer Observatory) 2020 report on the estimates of cancer incidence and mortality among the people of 185 countries (produced by the International Agency for Research on Cancer), female breast cancer (BC) has been identified as the most commonly diagnosed cancer (11.7%) and is the fifth leading cause of cancer mortality worldwide [1]. The same report suggests that among women, BC accounts for 1 in 4 cancer cases and for 1 in 6 cancer deaths, ranking first for incidence in most of the countries (159 of 185 countries). Through numerous studies, researchers found that early detection can significantly reduce fatal outcomes, and awareness is one of the keys to early detection [2]. Spreading awareness is a challenge for different societies for various reasons, and researchers have been working on identifying those for a long time. In this work, we assess the barriers to mass awareness (and thus far early detection) of BC among people in 2 very densely populated but culturally similar and geographically neighboring areas—Bangladesh and West Bengal, India.

Bangladesh (area: 57,321 square miles) and West Bengal, India (area: 34,267 square miles) are 2 territories in South Asia. Bangladesh’s official language is Bengali, and the mass people primarily use the Bengali language for daily conversation. In total, 8% (91,276,115/1,210,569,573) of the Indian population speaks Bengali and mainly resides east of the country, with a concentration in West Bengal [3]. The population density of Bangladesh is 1328.68 people per sq km (collected from the web), and in West Bengal, India is 1028 people per sq km (according to their 2021 census). The deep sociocultural linkages between the Bengali linguistic community in eastern India and Bangladesh remain stable even after India’s partition and independence in 1947. The unemployment rate in Bangladesh is 5.23%, and in West Bengal, it is 5.2% (as of June 2022). A total of 70.54% (64,385,546/91,276,115) of people from West Bengal follow Hinduism, and 27.01% (24,654,825/91,276,115) follow Islam. In Bangladesh, 91.04% (150,360,405/169,828,921) are Muslims, and 7.95% (13,130,109/169,828,921) are Hindus. The life expectancy in West Bengal, India, is 71.2 years [4], and in Bangladesh, it is 73.57 years [5]. The approximate sex ratio (as of 2022) in Bangladesh is 102.12 males per 100 females, whereas in West Bengal, it is 105 males for every 100 females. The average literacy in Bangladesh is 74.91% [6], and in West Bengal is 76.26% [6]. The commonality in the history and sociocultural environment of eastern India and Bangladesh has created a pseudo-homogeneous society where language is one of the fundamental elements in this connectivity. Hence, our work focuses on understanding attitudes, perceptions, and behaviors around BC-related issues in these 2 densely populated areas.

Unfortunately, there are no available sources of comprehensive epidemiological, pathological, and outcomes data for patients with BC [7,8] for the chosen regions; so, we are using the closest information from the GLOBOCAN 2020 report [1] to estimate the incidence and mortality rate of BC in this part of the world (Table 1). The factors contributing to the rising incidence and mortality of BC in low-income countries such as Bangladesh and West Bengal, India, are multifaceted. It is important to acknowledge that the solutions required for addressing these challenges differ significantly from those implemented in high-income countries [9]. Therefore, adopting models or policies directly from high- or middle-income countries, which have demonstrated success in tackling BC, may not be applicable in this context.

**Table 1:** Age-standardized incidence rate and age-standardized mortality rate of breast cancer in India and Bangladesh (GLOBOCAN 2020 report) and some relevant sources like the World Health Organization.

Scope	Age-standardized incidence rate in 100,000	Age-standardized mortality rate in 100,000
World	47.8 [1]	13.6 [1]
South Central Asia (Bangladesh and India are geographically part of the area)	26.2­­ [1]	13.1 [1]
West Bengal, India	25.2 [8]	13.62 [10]
Bangladesh	21.4 [8]	10.69 [11]

Despite being classified as least developed countries, Bangladesh and West Bengal, India, are home to several highly equipped and specialized cancer hospitals, some for BC only; for example, the National Institute of Cancer Research and Hospital (Dhaka, Bangladesh) or Tata Medical Center (Kolkata, West Bengal, India). These institutions, staffed with highly skilled and experienced surgeons, oncologists, and gynecologists, offer mammograms, CT (computed tomography) scans, and other modern screening and medical facilities. However, advanced medical facilities are scarce in rural and suburban areas, and the capacity of existing institutions often falls short of meeting the demand. In addition, there are also significant uncertainties regarding the overall cost of treatment, the duration of care, mental health support, and gaps in preventative measures. Given this context, our goal is to identify key gaps in existing approaches to raising awareness about BC and addressing the barriers that prevent people from seeking preventative care and timely treatment. By targeting these blind spots, we aim to make meaningful changes that promote early detection and improve access to necessary medical services.

In this work, to understand the barriers to BC awareness, we conducted an open-ended interview with the residents from both regions about BC awareness (to assess their current state of knowledge, attitude and readiness, awareness, and so on) After carefully analyzing our interview data, we highlighted some common obstacles in both regions or societies, by grouping them into different societal levels such as individual, family, local society, health care system, and state or country. In the upcoming sections, we discuss the study design (Methods), the highlighted barriers with some root causes, and suggested solutions (Results); then, we suggest how technology and social media can play a crucial role in resolving some of those effectively (Discussion).

## Methods

### Study Design

The main objective was to evaluate the general knowledge and awareness of the participants regarding BC, identify sociocultural factors that could hinder mass awareness campaigns, and examine health care disparities and systemic issues. We conducted individual interviews with the participants using a semi structured questionnaire that included a variety of specific questions as well as 1 open-ended question (Table 2). The participants’ responses included personal experiences and anecdotal stories.

**Table 2:** Our questionnaire.

Category	Questions
Awareness	People of which age range do you think are at high risk of getting breast cancer? What are the most common symptoms of Breast Cancer? What are the common misconceptions about Breast Cancer? Are there any media-related programs that have provided information regarding Breast Cancer or Breast Cancer diagnosis or progression of the disease? Example: Television talk shows, YouTube, WhatsApp, Facebook groups, or movie or television shows Are you aware that breast cancer can affect anybody regardless of their sex and gender classification? If not, what gender and sex demographic do you think have the possibility of getting Breast Cancer?
Self-diagnosis	Do you know and voluntarily do the Breast test yourself at home?
Post-diagnosis support	What were the support mechanisms (nonpharma) provided to you? For example, mental health or NGO support, nutrition, or self-care. Was the financial impact of the proposed treatment plan discussed with the doctor?
Open-ended	Is there anything you would like to share about your journey or experience with Breast Cancer?

^a^NGO: nongovernmental organization.

### Participant Selection and Recruitment

**Figure 1:**
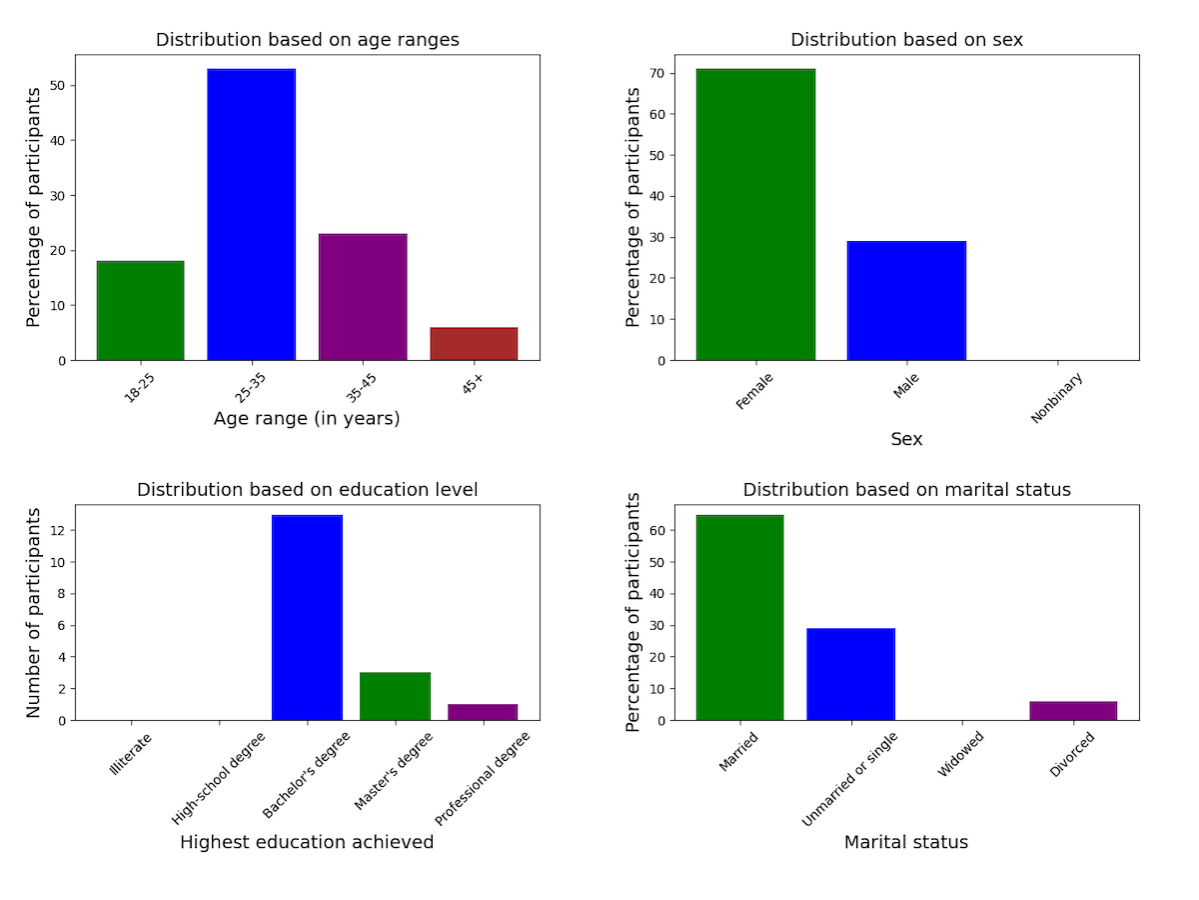
Distribution of participants.

We recruited participants through snowball sampling [12], using mutual connections and social media platforms such as Facebook (Meta). Semistructured interviews were conducted with 17 participants from West Bengal and Bangladesh. All our participants were above the age of 18 years, and recruitment was focused on participants who resided (at the time of study) in the 2 regions mentioned above. Our participants primarily identified as female, with an average age of 25-35 years, and most were married (Figure 1). The participants selected the language during the interview; most spoke Bengali. The researchers conducted the study through Zoom (Zoom Video Communications), and the audio recordings were transcribed and translated into English for data analysis.

### Demographics

We aimed to recruit a representative sample of participants from diverse educational and financial backgrounds from the region, as socioeconomic status and education play a huge role in the participants’ access to health care in the region. Also, a cultural stereotype related to educational qualifications is widely propagated, relating educational accomplishments to open-mindedness, access to health care, and overall “success” in life [13]. Our participants self-declared their financial status as middle- and high-economic classes and were all in professions such as business, architecture, social development work, or education. Most of our participants had completed their undergraduate degrees (n=13), few had graduate degrees (n=3), and some were currently working toward a professional degree (n=1). The socioeconomic status of our participants is important in the larger context of interpreting the results and will be discussed further in the upcoming section.

### Data Collection and Analysis

**Figure 2:**
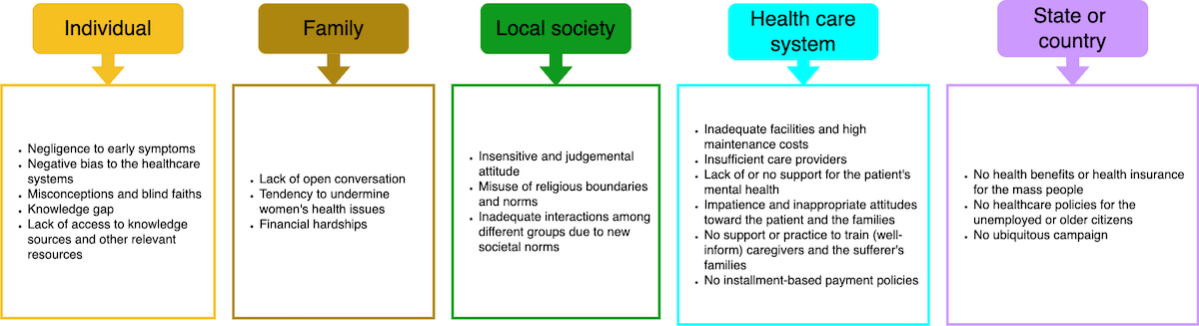
Outcome of our qualitative data analysis: major barriers to breast cancer awareness in the region.

We transcribed the interview audio recordings, which were between 15 and 45 minutes long, and translated into English. Our qualitative data analysis follows a tiered approach and an inductive methodology [14,15] to generate themes. In tier 1, the initial coder examined the transcript identifying recurring themes, clustering them based on similarity, and using the descriptive coding methodology to label them. This clustering formed the foundation for subsequent analysis. Moving to tier 2, a second coder independently reviewed the clustered themes and descriptive terms associated with them, providing validation by either corroborating existing clusters or proposing new ones as necessary. Finally, tier 3 involved collaborative discussions between both coders to assess the relevance and significance of the clustered themes and descriptive terms associated with them. Through iterative deliberations and consensus-building, the final classification of themes was determined (Figure 2). As our interviews were semistructured (where the participants had the freedom to take the narrative and provide more information beyond a fixed set of questions), this method of generating themes bottom up and assigning descriptive terms to it allowed us the flexibility to show nuance and highlight subthemes. Both coders were familiar with the language spoken by the participants (Bengali) to parse through the linguistic, cultural, and social significance of the data generated during the interview sessions.

The hierarchical representation of our themes and subsequent categories (Figure 2) is inspired by the presentation of the barrier tree [16] developed for a mobile-based BC monitoring study for the people in rural Bangladesh around 2012. Notably, several factors (such as feeling shy, fear, lack of familiarity, undermining women problems, religious belief, shared beliefs and practices, communication problems, scarcity of doctors, inconsistent patient data, long-term monitoring, and poverty) identified then are still highly relevant and came up during our analysis. The distinctions between both works are listed below.

First, most of our participants are city dwellers, but they shared stories and experiences from different parts (city, suburb, or rural) of the country or state during their interviews. Haque et al [16] studied in rural setups about 1.2 decades ago.

Second, we present the barriers by clustering them into 5 socioeconomic themes (individual, family, local society, health care system, and country or state). Haque et al [16] presented their findings into categories like identification and disclosure, achieving treatment, continuation of treatment, environmental issues, and user issues.

Third, while many research articles in the domain of BC primarily adopt a medical science perspective, such as the study by Haque et al [16], where the team visited a clinic or hospital to gather data and generate a barrier tree, our approach diverges by examining BC through a societal framework. By doing so, we aim to offer a comprehensive analysis that encompasses both the medical and societal impacts of BC. This approach allows us to explore the same problem from different lenses, shedding light on the multifaceted challenges and implications of BC beyond the purely medical aspect.

Finally, as a novel addition to the field, we delve into the mental health relevancy of patients with cancer and their caregivers, a crucial aspect that was not addressed in the other work.

We discuss the outcome of the qualitative analysis in the next section.

### Ethical Considerations

The institutional review board of New College of Florida approved the study (Protocol #22-037), and we strictly followed all protocols to ensure data privacy and integrity, demonstrating our commitment to ethical standards. For the study, participation was voluntary; participants did not have to participate and could stop at any time. There were no penalties or loss of benefits or opportunities if the participant did not participate or decided to stop once started. Their decision to participate or not to participate did not affect their job status, employment record, employee evaluations, or advancement opportunities. The participants did not receive any benefit from the research team. There was no cost to participate. This research was considered minimal risk. Minimal risk means that the study risks are the same as those in daily life. 

## Results

The results of the data analysis are discussed in 2 phases; first, barrier identification and second, awareness raising.

### Barrier Identification

**Figure 3:**
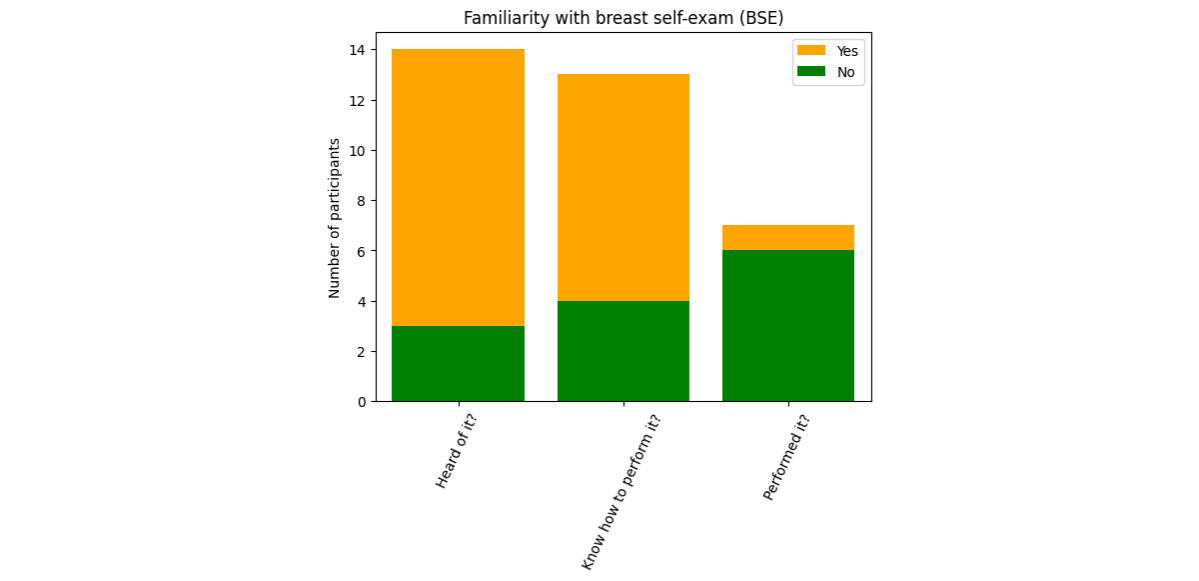
Familiarity test for “breast self-examination.”.

The majority of our participants were aware of BC’s symptoms, possible treatment mechanisms, costs, survival rate, and so on. During the interviews, they were able to list most of the significant symptoms of BC, like deformed breasts or nipples, lumps in the breast and armpit, discharge of pus or liquids, irritation, flaky breast skin, irregular breast pains, overall fatigue and weight loss, and so on. All but 3 participants knew of at least 1 person who has or had BC—either in their own or extended family, in the neighborhood, or in a friend’s family—indicating how predominant this disease has become (Tables 3 and 4). A total of 14 participants “had heard of” self–breast-examinations, 13 participants “knew how to” perform self–breast-examinations, and 7 participants “had performed” self–breast-examinations (Figure 3). A greater than 50% decrease in the number of participants who had conducted self–breast examination is noteworthy.

**Table 3:** Number of responses to the question: “How many patients with breast cancer do you know of?”

Percentage of participants	Number of patients or survivors with BC^a^
18	0
35	1
35	2
6	3
6	4

^a^BC: breast cancer.

**Table 4:** Patients with breast cancer in immediate family.

Percentage of participants	Number of patients or survivors with BC^a^ in immediate family
59	0
24	1
17	2

^a^BC: breast cancer.

The following sections share the barriers we highlighted from our data. Since the barriers often have cause-and-effect relationships and circular causality, we will not present them according to the identified societal levels like in Figure 2; instead, we would discuss them under the same section or title if they mostly appeared together during the interviews.

#### Tendency to Neglect Early Symptoms and Knowledge Gap

Our survey participants agreed that although BC is well-known, many people are unaware of the symptoms and often miss early signs. Specifically, when trying to identify symptoms in themselves, they either don’t know what to look for or tend to disregard early signs of discomfort. For example, 1 participant stated:

One day, my mom had severe stomach pain. We took her to the doctor, who asked us to complete a USG of her abdomen as the doctor suspected that my mom had a tumor in her stomach. While doing the USG for the tumor, the doctor detected that she had gotten breast cancer, not a tumor…… it was already a late detection. It’s at a deadly stage already. The treatment would have been much less painful if she had been diagnosed earlier.

b007

A similar story also stated the reluctance to pay heed to early symptoms or disclose them:

Though she (a neighbor) suspected it, she did not disclose it initially. When the pain became unbearable, then only she went to the doctor. She went to the doctors in the middle stage (not the early stage).

b011

One of my aunts (a family friend) had been diagnosed with breast cancer. However, it had already spread to the throat and jaw when she was diagnosed. So, the doctors could not remove/cure it, and she passed away.

b008

These responses reflect the results Amin et al [17] found in a survey that the research team conducted in Bangladesh in 2020; out of the 9 symptoms of BC, approximately 50% (N=500) of participants were unaware of 5 or more symptoms.

#### Misconceptions

Although none of our participants were medical professionals, our participant pool consisted of individuals who had all completed at least an undergraduate degree, were socioeconomically affluent, and were self-professed, open-minded individuals. Due to their educational backgrounds, we assumed that the number of misconceptions about BC would be lower than other groups. However, our participants had several deep-rooted misconceptions about BC, which are listed below.

First, it can only afflict females. Surprisingly, 10 out of 17 participants (59%) believed BC could only afflict females. The rest of the participants agreed BC occurred, irrespective of sex.

Second, it is more likely to afflict married women and those who have recently become mothers. Several participants stated that in married women, especially after giving birth to a child or during the breastfeeding period, it is highly likely to get inflicted with BC.

Third, it is more likely to afflict older women. Several participants responded that BC only affects older women; when probed further, they believed that the age range between 25 and 45 was not particularly susceptible to BC. Reports have suggested that Indian women in their early thirties till fifties are at considerable risk of developing BC, and the incidence risk increases till its peak when they reach 50-64 years of age. Furthermore, 1 in 28 Indian women are likely to develop BC during their lifetime [18-21]. It is more (1 in 22) for urban women than for the rural group (1 in 60). Amin et al [17] report similar statistics for Bangladesh (22.5 per 100,000 women and mean age being 41.8 years and 56% of the diagnoses were for reproductive-age women).

At the end of our interviews, we informed our participants that (1) BC is statistically more dominant among females but can afflict anyone, irrespective of gender and (2) there are various types (metastatic, inflammatory, and so on) of BCs that afflict people from various age ranges, so directly linking it to a particular age, marital status, or maternity may not be the best idea.

These factual misconceptions and knowledge gaps about BC are major factors related to the diagnosis of BC at an early stage. Our participant responses reinforced the need to disseminate accurate information to raise awareness.

#### Social Stigma and Lack of Open Conversation

First, beauty and breasts—judgmental attitudes. In addition to being an organ, breasts play a significant role in defining femininity and women’s role in South Asian cultures. The fear of the disease attacks the fundamentals of female identity as a woman’s breast conjures her sexuality and her capacity to nurture [22]. In patriarchal societies, such as Bengalis in West Bengal and Bangladesh, irrespective of educational or financial gains and social liberties through the years, women are perceived to have the roles of mothers, wives, or homemakers, whereas men are mostly seen as primary breadwinners of the family [23].

According to a report by a Pakistani Non-Governmental Organization, Aurat Foundation and USAID (United States Agency for International Development; 2016), in South Asian cultures, feminine traits include emotional, nurturing, passive, good homemakers, fair-skinned, without body or facial hair, long-haired, with a narrow waist, and sexually submissive. In contrast, masculine traits include being assertive, primary breadwinners, risk-taking, rational, and brave [24]. The ultimate danger of this notion to a Bengali (or maybe many people from other parts of the world) is that they feel one becomes “lesser feminine” if one has any breast health-related issue. On top of that, many people assume that BC’s ultimate aftereffect is mastectomy, if not death, which adds a new level of stigma [25,26].

When I was very young, I learned that one of my friend’s mom had breast cancer. At that time, my parents were very conservative; they would not open up in front of other family members/us. I learned later that doctors had to remove her breast. But the good thing is, she recovered totally, and it’s been 20 years. She has an everyday life. I believe she received good treatment. Every treatment doesn’t go wrong!

b009

Of the few breast-cancer and other cancer survivors I know of, I say it is common for breast cancer patients to feel humiliated or more mentally upset than other cancer patients. …… I don’t notice any initiative in our society in this matter; neither is it commonly seen to provide some counseling for the patients for overcoming/fighting cancer.

b005

For several reasons, anyone with BC is reluctant to share their experiences. In many cases, patients with BC suffer from depression [17] due to a lack of a support system to debunk the body image and societal myths. The image of an ideal woman propagated in the media (television or social media) reinforces the narrative that is sometimes unhealthy to a person’s body image [27]. Particularly the lack of influential media personalities sharing their stories of struggle and recovery or the absence of characters that represent the struggles of patients or survivors of cancer in television shows or media portrayals impacts the perception of the journey and struggles.

Our participants echoed these sentiments in their statements:

There is a taboo around breast cancer, regardless of class and status. People do not talk about it. We learn about it later, like once someone has recovered or died of breast cancer. Mostly, no one shares their journey with breast cancer.

b014

She (a breast cancer survivor) used to talk to me and hang out with our family. However, she and I never talked about it. Neither I asked, nor she opened up. If it is not very necessary, we will not share. That is the mindset.

b012

The term cancer comes with a connotation of impending doom and death; however, the significantly higher chances of patients surviving cancer because of early detection strategies and awareness are not emphasized at all [28]. When it comes to the 5-year overall survival, a study reported it to be 95% for stage I patients, 92% for stage II, 70% for stage III, and only 21% for stage IV patients [29]. It is important to highlight these statistics widely to encourage early detection and awareness. Survival rates of patients with BC are lower compared with Western countries, primarily attributed to early age onset, late-stage detection and delayed initiation of definitive management, and inadequate or fragmented treatment [28].

Second, cultural sensitivities. In addition to not expressing discomfort, awareness campaigns in India and Bangladesh must be wary of the strict cultural, religious, and moral censorship, which does not allow the posting of revealing images of breasts. People, in general, are brought up in an environment where they do not feel comfortable discussing sexual health or women’s health concerns, including BC. The term “breast” is perceived through the lens of hypersexualization and hence comes under deep scrutiny from moral and religious sensitivities. As researchers embark on developing awareness campaigns, it becomes crucial to exercise cultural sensitivity, especially considering the conservative nature of the audience in these regions. Being mindful of cultural nuances is essential to ensure that the true essence of the campaign is not overshadowed by moral policing or societal restrictions. It is important to point out that women’s health or sexual health and the term breast are not taboo; we cannot undo decades of cultural teaching and biases in a day. Hence respecting the norms and designing awareness campaigns around them would be a more sensitive approach.

She (my paternal aunt, who was a teacher) had lung infections and troubled breathing due to breast cancer, but the doctor tracked it back to breast cancer after doing several medical tests; she was never open about the symptoms (or discomforts with her breasts) upfront to the doctor.

b001

People are not comfortable talking about it. Since I am the son, my mom also never shared about her symptoms or sufferings.

b007

Third, women’s health care is a necessity, not a luxury. Irrespective of South Asian patriarchal societies or more progressive Western societies, a woman’s role in society, economy, and family cannot be ignored. The roles of caregiver, parent, and wife for a rural woman with added burdens of maintaining appearance and body image in the case of an urban woman all contribute to the issues that the contributions women make to the world are “nonproductive” or “nonessential.” This perception sometimes from women themselves (in some cases, the family members) leads to the health concerns of women being ignored or underprioritized [23]. This includes a lack of attention to proper nutrition, timely intervention, care after diagnosis, and adequate mental health support. This practice or habit stood out as one of the major reasons why female patients often get a late detection of health-related problems.

We wait and hope that the discomforts will disappear automatically after a while – so we tend to linger. I have noticed this among my friends and family (especially females), including myself; we tend not to go to doctors so easily. I am not sure if it’s a cultural thing! But it is less about financial conditions and more about awareness.

b014

Certainly, that mindset is changing gradually. Nonetheless, there is a need for increased awareness in this particular area:

A woman in the family will likely delay her treatment as she thinks it’s the least important issue and is probably putting financial pressure on the family. But sometimes, they forget that delayed treatments may invite worse consequences.

b003

#### The Disconnect Between Health Care Models and People

Although none of the existing health care models worldwide are perfect, in the context of a deadly disease like BC and health-related supports, we noted the flaws, inconsistencies, and lack of patient-centered policies in the regions.

First, lack of a yearly health examination policy. Unlike in the United States or other parts of the world, Bengalis usually do not go for yearly health examinations, or it is not a common practice among doctors to prescribe yearly health checkups. The lack of adequate resources, such as available medical professionals, provider-centric consultations, and a symptom-based health care model, is partially responsible for this issue [30]. The region requires immediate advocacy for a robust primary health care system and the use of existing community care efforts to enhance awareness.

Second, out-of-pocket medical expenses. Health examinations are expensive, and it is unusual for the region to support health insurance employers [31]. There is no system of national health insurance in Bangladesh [31]. People whose employers cover health benefits also must pay to the facilities out of pocket first and then later ask for a reimbursement. This cost barrier leads to patients not seeking treatment early on.

Third, lack of integrated service and long-term plans. Those on tight budgets usually have to prioritize the price over reliability and integrated service available to few modern (and more expensive) health care facilities. Patients and their family members are often not fully aware of the overall costs (long-term plans alongside short-term plans) and risk factors so that they can make informed decisions.

Talking to the patient and their family about the situation and possibilities is crucial. Only then can we make an informed decision. But if the doctor dictates the options, the patient’s family has to follow the order, no matter how expensive or if they have to lose their property in arranging the financial support.

b009

Fourth, open conversation between patients and the doctors. Due to various reasons, including high volume of patients, doctors seem to spend much less time with the patients, which has been a consistent story we heard from our participants.

The more renowned the doctors are, the less interactive they are, or the less compassionate they are. There is much less scope and the chance of openly conversing with them.

b014

Fifth, insensitive and judgmental commenting. Medical professionals lack adequate training when addressing patients and their concerns. A dearth of empathy compounded by the enormous patient loads leads to negative interactions with the health care providers, and patients or family members do not feel comfortable asking essential questions. An insensitive comment may cause severe damage to the patient’s (or their caregiver’s) confidence and motivation. Effective doctor-patient communication is determined by the doctor’s bedside manner, which patients judge as a major indicator of their doctor’s general competence [32,33]. Doctor-patient communication skills are based on verbal and nonverbal communication, where 22% is transferred through voice tone, 55% through visual cues, and 7% verbally [34]. Lack of training to demonstrate effective behavior leads to scarring patient outcomes, one such is listed below:

I remember an oncologist’s comment to his patient (lung cancer survivor, 65+ years of age) regarding the side effects of Chemo/radiotherapy: you don’t have your natural hair anymore! So why worry about that?

b003

Sixth, the doctor’s gender. BC disproportionately afflicts the female population, and since it concerns an intimate body part, it is common among the patients and their family members to withhold the treatment until they find a female specialist; in short, they prioritize the doctors’ gender over professional expertise [32]. In a cross-sectional study conducted with 1257 female patients in Udaipur, India (August 2014), by Nagarajappa et al [32] found an overwhelming 85% (1069/1257) of the respondents said yes to the question: “I would always prefer being treated by a doctor of my gender.” In some other similar studies conducted in 2019, researchers found that the most important attributes of maternal health care facility choice for Bangladeshi women were consistent access to a female doctor, the availability of branded drugs, respectful provider attitudes, and a continuum of maternal health care [35,36].

My aunt and uncle delayed the treatment as they were hesitant about choosing a doctor; they were looking for a female doctor, but there were not many female breast cancer specialists in Bangladesh during that period. My uncle (my mother’s cousin) had hesitation. Had she been diagnosed earlier, she would have had a better chance of survival. She was a survivor, but her entire family suffered greatly due to the delay. My uncle later regretted his decision. I remember him expressing that to my mother (his sister).

b003

Although 61.1% (198/324) of medical students in India identify as female, a study by Bajpai et al [37] in 2020 found that men led 67.3% (218/324) of oncology teams in India. These numbers are similar to the numbers in the United States, where out of 667 female respondents, 442 identified as academic oncologists versus nonacademic ones [38]. This gender disbalance among physicians leads to patients not seeking timely medical care.

Seventh, lack of trust. Many people in the region believe that:

…an institute (hospital and caregiving center) that is capitalist in nature cannot have people’s best interest at heart.

b016

This may play a significant role in people’s hesitancy to seek health advice unless necessary.

#### Mental Health Support Infrastructure

All participants expressed concerns about lacking mental health support systems. Most reported that:

- family members and caregivers are never made aware of the patient’s mental health, its relevance, and how to handle them [26].
- “I didn’t know and never thought mental health could be impacted directly by these (or any) diseases.” [b007]
- there is no infrastructure to support the mental health of patients suffering from terminal or deadly diseases.
- “Mental health is considered taboo, more significant of a taboo than breast cancer. People don’t usually talk about it. Doctors also don’t do a good job in this regard. Providing professional mental health support is not common in India/West Bengal to battle cancer.” [b016]
- “Since cancer is associated with a lot of uncertainty, fear, and stress, the moment patients get diagnosed with cancer, they must get counseling. Mental support from proper channels can help patients gather the strength and courage to defeat it.” [b010]

#### Role of Media

Media has always played a significant role in spreading awareness among the general population on various issues. We wanted to learn how our participants felt about the role of media around them in spreading BC awareness. A survey conducted by YouGov Global Media in India identified that in 2022, 59% of Indians under the age of 24 years depended on websites or web apps as their primary media source, whereas people above the age of 45 years depended on television (live or nonlive broadcast) as a primary media source [39]. In Bangladesh, USAID conducted a similar survey and found that 46.6% of respondents mentioned television, and then 33.1% said the internet was a primary source of information media [40]. Similarly, in India, 60% of the population in the age group of 45 years and above depend on television for news, whereas younger audiences tend to lean toward updates from social media instead of watching traditional television news (which broadcasts news at specific hours).

Social media and traditional media (television and newspapers) can substantially spread awareness among people. We wanted to understand whether the media’s contribution to raising awareness was limited to a date or time (eg, Breast Cancer Awareness Month) or whether there is any consistent campaigning.

Our participants have reported seeing numerous posts, symbols (such as the pink ribbon or black screen), and talk shows on specific days, weeks, or months of the year (such as during Breast Cancer Awareness Day). However, these efforts are transitory. They come and go; many posts are left unattended, unread, and unfollowed, and many programs and talk shows are left unwatched. The lack of consistent programming on any platform that regularly sends out relevant messages throughout the year leads to reduced engagement and interest.

I have seen many programs aired on the national television of Bangladesh, but I believe that is not sufficient…. I haven’t noticed any program regularly on this topic. If we talk about social media, I don’t see enough posts relevant to this topic.

b004

People share (even if that’s at a minimal level, especially on special occasions like Breast Cancer Month). But others may not have time to read it carefully due to their busy schedules. I sometimes see such posts but don’t have time to read them thoroughly. I feel until it hits someone, they are likely to ignore (or blindside) it.

b003

**Figure 4:**
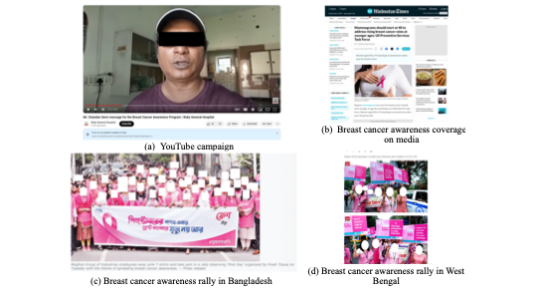
Media coverage, rallies, and campaigns.

Most BC-related posts (eg, Figure 4) on media platforms include either a pink bow [41,42] or wearing the color pink at campaign events [43,44]. To urban audiences’ mammograms are widely promoted by health care professionals or organizations [41,45].

The absence of relevant information about the disease, prognosis, and symptoms is stark and leaves the audience wondering how to perceive this awareness campaign. Often, momentum is lost, and true engagement is not achieved, leading to lackluster awareness numbers.

We see advertisements relating to breast cancer, sometimes on the television or elsewhere, or billboards, but then it is very short and crisp. And it just says that we should be aware of breast cancer, but then what to be aware of it is never mentioned anywhere.

b017

### Awareness Raising

**Figure 5:**
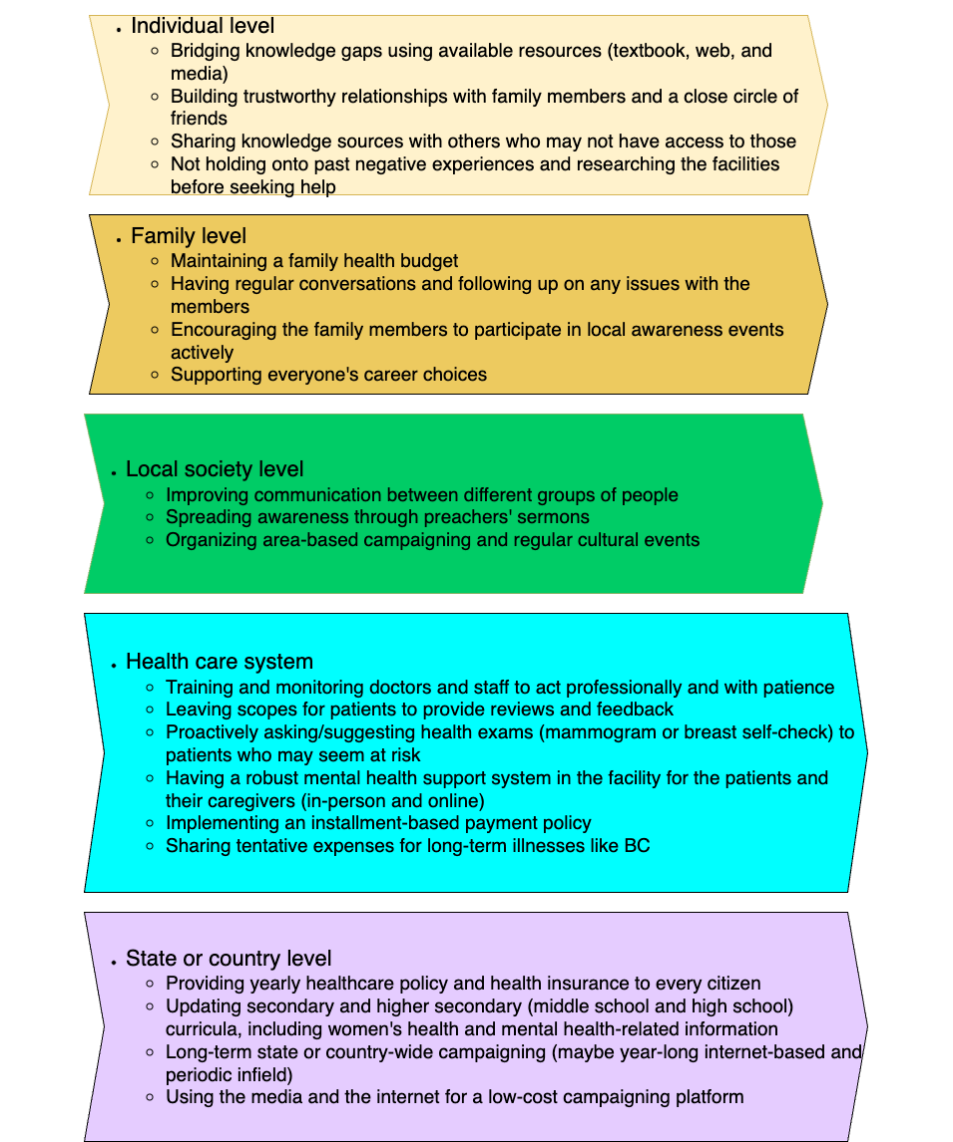
Our recommendation to help raise awareness. BC: breast cancer.

Many of the findings are codependent and mutually recursive. For example, lack of trust in health care systems in the area and financial hardships implicitly push people to neglect early symptoms of deadly diseases; lack of open conversation and social stigma block them from reaching out for mental health support, to name a few. Our discussions with the participants highlighted several means to help spread mass awareness in the regions. In this section, we present the summary of our suggestions to achieve mass awareness through Figure 5 and briefly discuss them right after.

#### Designing a Yearly Health Care Routine for Everyone

Society must believe that early diagnosis can help increase survival and reduce suffering. We need to build the practice of going through a yearly checkup.

b008

Given the population, unemployment, and other issues, achieving mass success in building this practice will require support and cooperation from international organizations (such as the World Health Organization) and local governments.

#### Spreading the Voice of Survivors

We often hear words from specialists (doctors and scientists) on mass media (eg, Shastho Kotha [46]). Aside from their knowledgeable notes, the programs will draw attention to the mass scale if BC survivors’ interviews are included regularly. This will also reduce fear, hesitancy, and social stigma. When people see interviews of BC survivors belonging to the same community (age, gender, social status, and so on), it will help everyone to open up about similar issues.

I believe cancer changes our views and ways of life. We must break the practice of neglecting minor symptoms and not seeking medical advice. We need to improve trust and healthy communication between doctors and patients. Roughly 50% of the population is female. So, whichever cancer is more probabilistic to which population, they need to take it seriously. Financial hurdles should not be the first concern. It will be beneficial if there is a platform where people can talk about it and not treat it as something that cannot be discussed.

b016

Sharing the experiences of BC survivors as they navigate treatment plans, manage financial burdens, and embark on their journey toward recovery can play a crucial role in dispelling fear, reducing anxiety, and challenging the taboos surrounding BC. Narratives that illuminate the resilience of these survivors, highlight the support of their families, and shed light on the obstacles they overcome contribute to humanizing this daunting illness.

#### Using the Grassroots Health Workers

NGOs like Surjer Hashi Clinic, Shobuj Chata Community Clinic, etc., wildly succeeded in child immunization and birth control in Bangladesh. So, if cancer awareness (like breast and cervical cancer) is taken as an agenda by such organizations, I am hopeful it will succeed.

b003

If the health workers spread a simple flyer about BC with some visuals and depictions in Bangla during their weekly visits, it will help raise awareness in rural areas quickly.

Bangladesh has successfully rolled out the idea of condoms and contraception, though we are considered a very conservative society. Major religions of our land directly oppose the idea of birth control. Yet the success came not because of the paid advertisements or programs in media but because of sending medical advice to the grassroots level. So, I believe we can succeed in growing awareness about breast cancer if the Government takes such programs into the hand.

b005

#### Establishing a Collaborative Effort on Social Media

Bangladesh’s internet penetration rate stood at 31.5% (52.58 million/167.1 million) of the total population at the start of 2022 [47], and India’s 47% (658 million/1.40 billion) of the population has internet access [48]. India and Bangladesh have both shown steady growth in mobile cell phone service, too. Instant messaging apps such as WhatsApp (Meta; 487 million users in India and 40 million users in Bangladesh) [49,50]. We strongly believe these platforms can be used to spread accurate awareness messages to users, particularly if government health organizations partner.

**Figure 6:**
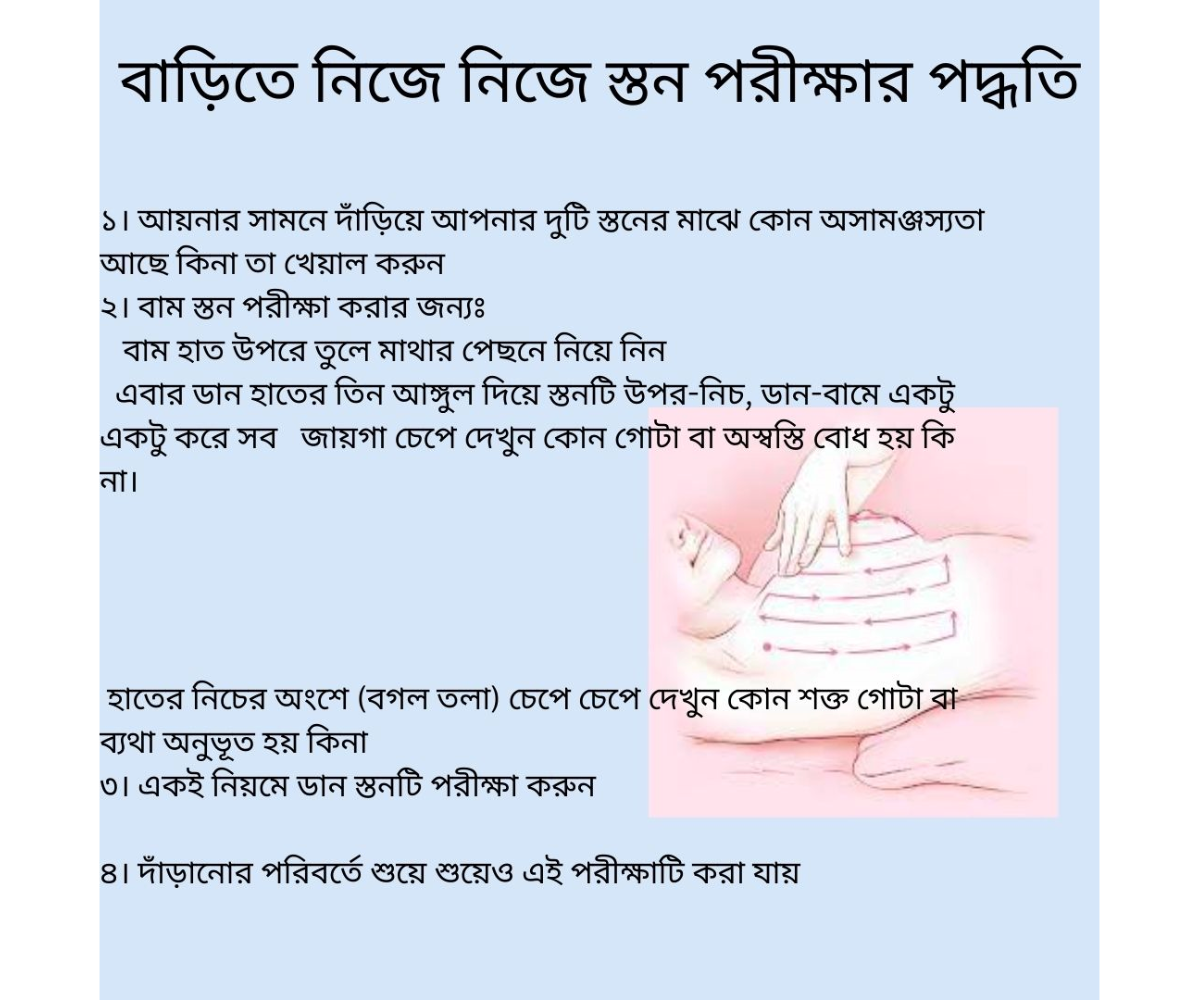
Proposed self-breast examination instruction flyer.

We suggest regularly preparing WhatsApp message broadcasts or mass forwards to groups, patients, or individuals from credible organizations such as local government branches (eg, zilla panchayat or district offices), medical associations (eg, Bangladesh Medical Association or Medical Council of India), or nonprofit organizations (eg, Sabuj Sathi). Sending these messages individually to users also avoids making anyone uncomfortable with the content, keeping in mind the social and religious sentiments or barriers in these regions. WhatsApp’s ubiquitous presence in the digital communication ecosystem of both countries removes additional app downloads or installations. We propose using WhatsApp forwards (illustrated in Figure 6) as a cost-effective, easy-to-understand, and time-efficient method for dissemination. However, it is essential that these messages are reviewed by an expert before distribution to ensure accuracy and reliability.

#### Inclusive Texts in Secondary and Higher Secondary (Middle and High School) Curricula

Many people in both regions finish their education till middle- or high-school level. Some inclusive articles (written with appropriate languages and cultural values in mind) regarding women’s health and the terminal and most predominant diseases in society will help spread awareness. The students and their families will gradually become aware; thus, many of the causes blocking the attention can be addressed thus far. Because textbooks can reach families where social media or entertainment media cannot.

I grew up in a village. I can talk to you from that context.… It isn’t easy to spread awareness in the villages. For example, I grew up in a Muslim family where watching TV was not allowed, as it is anticipated that kids will be misguided by watching cartoons or inappropriate programs. So, getting this knowledge through TV was/is challenging for many kids like me.

b006

Per our research, no story, article, or novel about BC is included in the secondary and higher-secondary curricula of Bangladesh and West Bengal, India.

#### Educating the Caregivers and the Family Members

Besides building infrastructure for mental health support, doctors and other associates must educate the family members and caregivers about the patient’s mental health and how to deal with it. We heard stories suggesting how a family can motivate a survivor to fight BC:

Someone very close to me has breast cancer. Initially, when it was diagnosed, the doctor prescribed her some medicine but barely communicated with her about her mental health. So, she melted down. After battling breast cancer for two years, she started self-motivating and regained her mental strength. She is a mother of two young kids, so that pushed her to keep going and not give up. She involved herself in activities like Yoga. Due to regaining mental strength, she started handling the treatment better (I am not suggesting that the cancer has disappeared, but she started driving it better).

b002

She (a relative) went into depression after the mastectomy. But since she has kids, she motivated herself. Her family also supported her very strongly. This support has helped her come out of the trauma. She is still following up with her breast cancer-related treatment. It is four years that she has been receiving treatments.

b011

## Discussion

In this research study, we aimed to understand the attitudes and perceptions of South Asian people, specifically people from Bangladesh and West Bengal, India, regarding BC. Our goal was to identify common barriers to raising mass awareness about BC in this population. In the results section, we discussed the significant barriers to BC awareness, which included a lack of knowledge and open conversation, negligence of early symptoms, negative bias toward health care systems, judgmental attitudes, and insensitive comments toward patients and their families, and financial hardship with limited options for financial support. We also provided suggestions to effectively raise mass awareness, including encouraging open conversations among family members and different groups, discussing relevant topics in social events and places of religious practices, integrating the topic into school curriculums, and disseminating accurate information through social and traditional media.

We present, in this work, the summaries of our findings in Figure 2 and Figure 5, where we clustered the critical points into 5 themes based on different societal levels—individual, family, local society, health care system, and state or country. Our findings highlighted the need for long-term planning and cooperation between people and the government, collaboration between national and international organizations, the use of media and education systems, and the importance of modern technologies and mass media in raising BC awareness.

Considering our dataset size (N=17) and the demographics of the participants, particularly their socioeconomic status and educational background, it is important to acknowledge that our findings are not fully representative of the entire population. Specifically, our dataset may not adequately capture the experiences of individuals living in rural areas, those below the poverty line, or those in the wealthiest brackets. These socioeconomic statuses certainly have disparate health care experiences and struggles. Our participant pool does offer valuable insights into the perspectives of the upper-middle-class segment of society and gives a general overview of barriers that should be further investigated.

For future work, it will be essential to extend this study to include participants from a wider range of economic and educational backgrounds. This broader approach will enable us to evaluate the generalizability of our findings and recommendations across diverse demographic groups and to uncover any critical issues that may have been overlooked in the current analysis. Such an expansion will contribute to a more comprehensive understanding of the nuances of this important issue and increase the applicability of our conclusions to a broader audience.

## Abbreviations

BC: breast cancer
CT: computed tomography
GLOBOCAN: Global Cancer Observatory
USAID: United States Agency for International Development
